# Supplementary material for: Optimization of Sample Preparation for Metabolomics Exploration of Urine, Feces, Blood and Saliva in Humans Using Combined NMR and UHPLC-HRMS Platforms
Source: Molecules. 2021 Jul 6;26(14):4111. doi: 10.3390/molecules26144111 (PMC8305469; doi:10.3390/molecules26144111)
Supplement: Supplementary file 1 [file molecules-26-04111-s001.zip › molecules-1280886-supplementary.pdf]

SUPPLEMENTARY DATA

Optimization of sample preparation for metabolomics exploration of urine, feces, blood and saliva in human using combined NMR and UHPLC-HRMS platforms

Cécile Martias[1], Nadine Baroukh[1], Sylvie Mavel[1],Hélène Blasco[1,3], Antoine Lefevre[1], Lea Roch[2], Fréderic Montigny[1], Julie Gatien[2], Laurent Schibler [2], Diane Dufour[1,3], Lydie Nadal-Desbarats[1], Patrick Emond[1.3,]

1 - UMR 1253, iBrain, University of Tours, Inserm, Tours, France

2 – ALLICE, Phenotyping station, Nouzilly, France

3- CHRU Tours, Medical biology center, Tours, France.

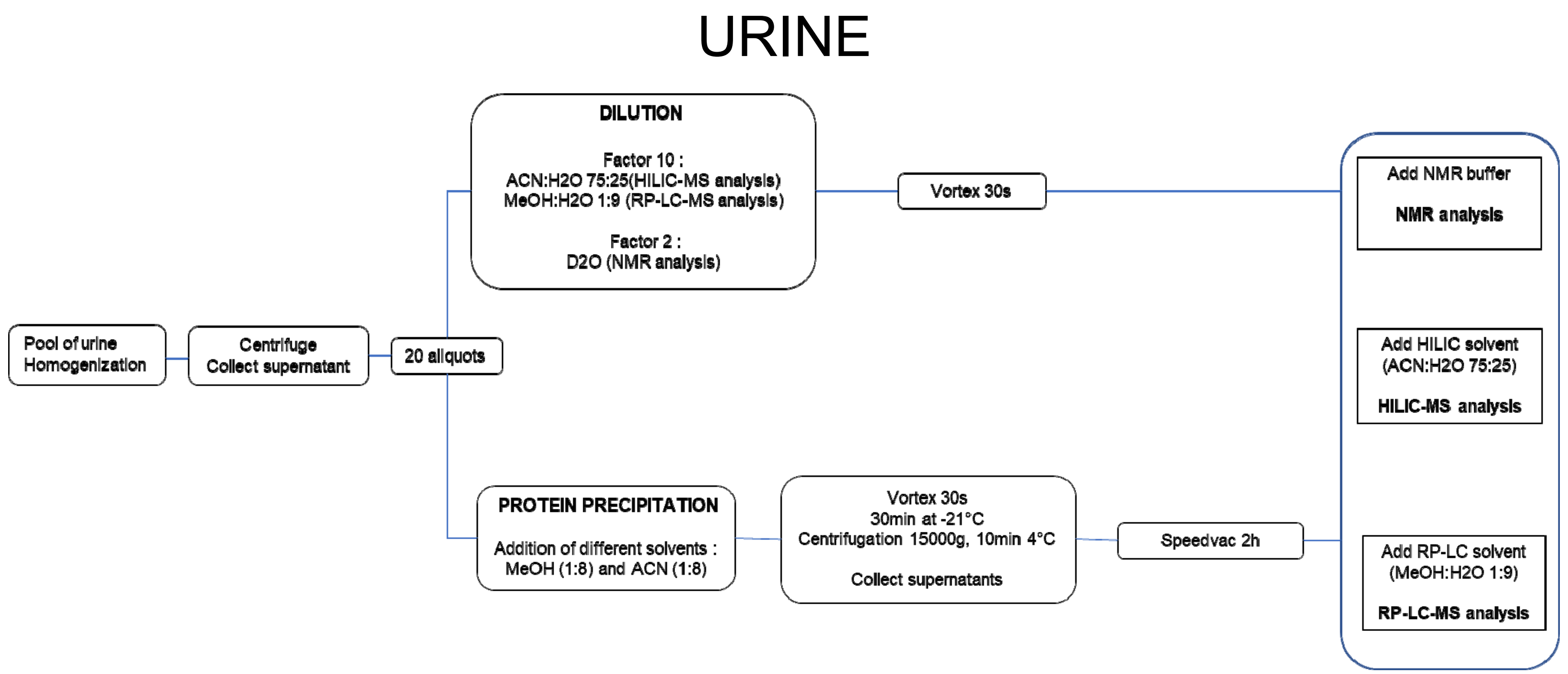

Figure S1: Schematic presentation of the preparation procedures applied on the pool of urine for the selection of the optimum protocol for the maximum metabolome coverage by LC-MS and 1H-NMR.

<table>
<tr><th rowspan="2"></th><th></th><th colspan="4">Numbers of buckets</th><th rowspan="2">CV – standard deviation</th></tr>
<tr><th>RP-LC POS</th><th>RP-LC NEG</th><th>HILIC POS</th><th>HILIC NEG</th><th>NMR</th></tr>
<tr><td>Urine :ACN (1 :8)
(n=5)</td><td>1976</td><td>1053</td><td>981</td><td>612</td><td>44</td><td>11% - 7%</td></tr>
<tr><td>Urine :MeOH (1 :8)
(n=5)</td><td>2335</td><td>1533</td><td>1030</td><td>830</td><td>45</td><td>9% - 6%</td></tr>
<tr><td>Urine :H_2O (1 :10)
(n=5)</td><td>2358</td><td>1532</td><td>1571</td><td>1052</td><td></td><td rowspan="2">9% - 6%</td></tr>
<tr><td>Urine :H_2O (1 :2)
(n=5)</td><td></td><td></td><td></td><td></td><td>46</td></tr>
</table>

Table S1: Number of extracted buckets in urine for the different extraction methods in ^{1}H-NMR and UHPLC-MS

		RP-LC	HILIC	NMR	TOTAL
Urine :ACN (1 :8) (n=5)	Number of metabolites	192	104	37	176
	CV<5%	18	0	9	27
	CV<10%	41	20	35	88
	CV<20%	64	59	36	132
	**CV<30%**	**72**	**81**	**36**	**145**
	CV % mean (min-max)	20% (1%-158%)	20% (5%-59%)	7% (2%-44%)	17% (1%-158%)
Urine :MeOH (1 :8) (n=5)	Number of metabolites	107	134	37	215
	CV<5%	41	1	23	64
	CV<10%	83	16	36	125
	CV<20%	98	72	36	172
	**CV<30%**	**102**	**114**	**36**	**201**
	CV % mean (min-max)	8% (1%-76%)	20% (4%-51%)	5% (4%-9%)	12% (1%-76%)
Urine :H_2O (1 :10) (n=5)	Number of metabolites	112	119		205
	CV<5%	47	7		82
	CV<10%	87	47		144
	CV<20%	99	99		186
	**CV<30%**	**105**	**112**		**197**
	CV % mean (min-max)	9% (0%-77%)	14% (2%-84%)		
Urine :H_2O (1 :2) (n=5)	Number of metabolites			37	
	CV<5%			32	
	CV<10%			35	
	CV<20%			37	
	**CV<30%**			**37**	
	CV % mean (min-max)			4% (2%-14%)	9% (0%-77%)

Table S2: Number of extracted metabolites and coefficient of validation in urine for the different extraction methods in ^{1}H-NMR and UHPLC-MS

**Pathway name**	**Urine:ACN (1:8)**		**Urine:MeOH (1:8)**		**Urine:H_2O**	
	**Hits**	***p-value***	**Hits**	***p*-value**	**Hits**	***p*-value**
Glyoxylate and dicarboxylate metabolism	7	1.86 E^{-3}	12	1.37 E^{-6}	9	4.17 E^{-4}
Phenylalanine metabolism	6	7.16 E^{-6}	6	3.58 E^{-5}	6	3.58 E^{-5}
Glycine, serine and threonine metabolism	7	1.52 E^{-3}	9	3.17 E^{-4}	8	1.65 E^{-3}
Citrate cycle	4	1.31 E^{-2}	6	9.16 E^{-4}	6	9.16 E^{-4}
Alanine, aspartate and glutamate metabolism	/	/	8	1.00 E^{-3}	/	/
Purine metabolism	/	/	13	1.2 E^{-3}	11	1.1 E^{-2}
Phenylalanine, tyrosine and tryptophane biosynthesis	2	2.01 E^{-2}	3	1.85 E^{-3}	3	1.85 E^{-3}
Glycerophospholipid metabolism	/	/	5	2.23 E^{-3}	/	/
Tryptophane metabolism	9	5.05 E^{-4}	9	3.68 E^{-3}	8	1.28 E^{-2}
beta-Alanine metabolism	/	/	6	4.46 E^{-3}	7	7.6 E^{-4}
Pantothenate and CoA biosynthesis	/	/	5	8.3 E^{-3}	/	/
Pyrimidine metabolism	/	/	8	9.46E^{-3}	/	/
Histidine metabolism	/	/	/	/	5	6.25 E^{-3}
Tyrosine metabolism	8	2.81 E^{-3}	/	/	/	/
Sphingolipid metabolism	3	1.38 E^{-2}	/	/	/	/
Synthesis and degradation of ketone bodies	2	3.22 E^{-2}	/	/	/	/
TOTAL	9 pathways		12 pathways		9 pathways	

Table S3: Metabolite set coverage highlighted by metabolites extracted (CV<30%) according to the three sample preparations. The number of hits and the p-values are shown for each preparation by metabolite sets.

BLOOD

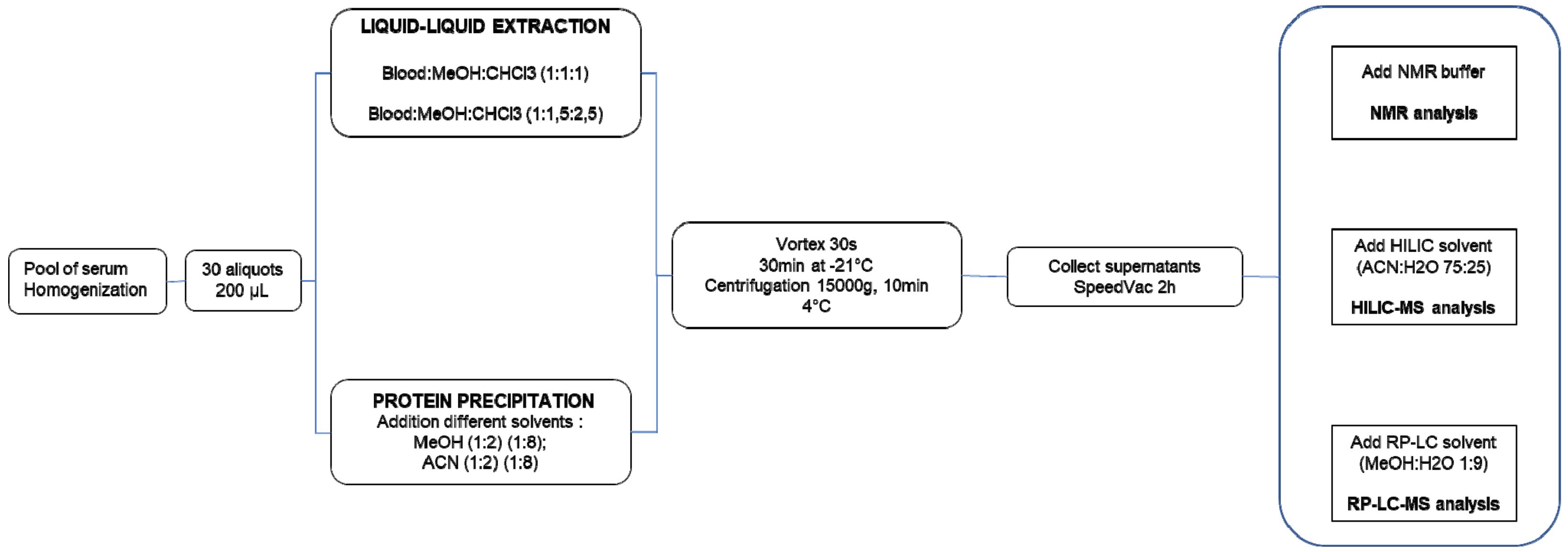

Figure S2: Schematic presentation of the preparation procedures applied on the pool of sera for the selection of the optimum protocol for the maximum metabolome coverage by UHPLC-MS and ^{1}H-NMR.

	Numbers of buckets					CV – standard deviation
	**RP-LC POS**	**RP-LC NEG**	**HILIC POS**	**HILIC NEG**	**NMR**	
Blood :ACN (1 :2) (n=5)	1233	1137	1624	2345	42	11% - 6%
Blood :ACN (1 :8) (n=5)	1173	997	1907	2373	37	13% - 6%
Blood :MeOH (1 :2) (n=5)	1285	1171	2018	2189	35	14% - 6%
Blood :MeOH (1 :8) (n=5)	1363	1167	2266	1908	42	13% - 5%
Blood: MeOH:$CHCl_3$ (1:1:1) (n=5)	996	1016	1049	1441	43	11% - 6%
Blood: MeOH:$CHCl_3$ (1:1.5:2.5) (n=5)	991	1020	1065	2019	42	14% - 6%

Table S4: Number of extracted buckets in blood for the different extraction methods in ^{1}H-NMR and UHPLC-MS

		RP-LC	HILIC	NMR	Total
Blood :ACN (1 :2) (n=5)	Number of metabolites	75	80	22	139
	CV<5%	45	3	4	51
	CV<10%	66	35	17	96
	CV<20%	71	66	19	122
	**CV<30%**	**72**	**74**	**20**	**129**
	CV % mean (min-max)	6% (1%-42%)	13%(4%-44%)	11% (4%-76%)	10% (1%-76%)
Blood :ACN (1 :8) (n=5)	Number of metabolites	66	82	22	135
	CV<5%	9	6	0	15
	CV<10%	24	36	4	58
	CV<20%	59	59	10	105
	**CV<30%**	**62**	**74**	**19**	**123**
	CV % mean (min-max)	12% (1%-39%)	15% (2%-74%)	20% (7%-37%)	14% (1%-74%)
Blood :MeOH (1 :2) (n=5)	Number of metabolites	76	88	22	147
	CV<5%	7	4	0	11
	CV<10%	50	28	4	75
	CV<20%	74	67	19	132
	**CV<30%**	**76**	**80**	**21**	**141**
	CV % mean (min-max)	9% (1%-29%)	16% (3%-77%)	14% (9%-40%)	12% (1%-77%)
Blood :MeOH (1 :8) (n=5)	Number of metabolites	89	88	22	157
	CV<5%	50	2	12	62
	CV<10%	73	36	19	107
	CV<20%	81	55	21	128
	**CV<30%**	**84**	**73**	**21**	**143**
	CV % mean (min-max)	7% (1%-60%)	19% (3%-123%)	6% (1%-33%)	11% (1%-72%)
Blood: MeOH:$CHCl_3$ (1:1:1) (n=5)	Number of metabolites	63	74	21	127
	CV<5%	9	5	16	30
	CV<10%	53	18	20	81
	CV<20%	62	52	20	112
	**CV<30%**	**63**	**61**	**21**	**119**
	CV % mean (min-max)	7% (1%-21%)	21% (1%-109%)	5% (2%-28%)	12% (1%-109%)
Blood: MeOH:$CHCl_3$ (1:1.5:2.5) (n=5)	Number of metabolites	60	76	22	126
	CV<5%	22	3	14	37
	CV<10%	54	26	18	88
	CV<20%	58	52	18	108
	**CV<30%**	**60**	**65**	**20**	**118**
	CV % mean (min-max)	6% (1%-28%)	17% (2%-56%)	10% (2%-75%)	11% (1%-75%)

Table S5: Number of extracted metabolites and coefficient of validation in blood for the different extraction methods in ^{1}H-NMR and UHPLC-MS

Pathway name	ACN 1:2		ACN 1:8		MeOH 1:2		MeOH 1:8		Blood: MeOH:CHCl3 1:1:1		Blood: MeOH:CHCl3 1:1.5:2.5	
	**Hits**	***p*-value**	**Hits**	***p*-value**	**Hits**	***p*-value**	**Hits**	***p*-value**	**Hits**	***p*-value**	**Hits**	***p*-value**
Tryptophan metabolism	9	4.15 E^{-4}	9	1.99 E^{-4}	9	5.55 E^{-4}	9	4.58 E^{-4}	/	/	/	/
Phenylalanine, tyrosine and tryptophane biosynthesis	3	7.63 E^{-4}	3	5.76 E^{-4}	3	8.55 E^{-4}	3	7.93 E^{-4}	3	5.52 E^{-4}	3	5.52 E^{-4}
Phenylalanine metabolism	4	1.81 E^{-3}	4	1.27 E^{-3}	4	2.09 E^{-3}	4	1.19 E^{-3}	3	1.31 E^{-2}	3	1.31 E^{-2}
Pantothenate and CoA biosynthesis	5	2.25 E^{-3}	4	1.08 E^{-2}	/	/	5	2.38 E^{-3}	4	1.02 E^{-2}	5	1.37 E^{-3}
Tyrosine metabolism	/	/	/	/	/	/	8	2.59 E^{-3}	/	/	/	/
Valine, leucine and isoleucine metabolism	3	8.98 E^{-3}	3	6.89 E^{-3}	3	9.99 E^{-3}	3	9.31 E^{-3}	3	6.62 E^{-3}	3	6.62 E^{-3}
Purine metabolism	/	/	8	2.00 E^{-2}	9	1.50 E^{-2}	9	1.28 E^{-2}	9	5.77 E^{-3}	9	5.77 E^{-3}
Ubiquinone and other terpenoid-quinone biosynthesis	/	/	/	/	/	/	3	1.34 E^{-2}	/	/	/	/
Pyrimidine metabolism	/	/	/	/	/	/	6	2.54 E^{-2}	/	/	/	/
Glycine, serine and threonine metabolism	7	1.30 E^{-3}	/	/	7	1.63 E^{-3}	/	/	6	3.88 E^{-3}	7	6.71 E^{-4}
Glycerophospholipid metabolism	/	/	4	2.90 E^{-2}	4	6.17 E^{-3}	/	/	4	3.62 E^{-3}	/	/
Glyoxylate and dicarboxylate metabolism	6	7.90 E^{-3}	/	/	4	9.50 E^{-3}	/	/	7	8.30 E^{-4}	6	4.61 E^{-3}
Sphingolipid metabolism	3	1.29 E^{-2}	/	/	3	1.43 E^{-2}	/	/	/	/	/	/
Alanine, aspartate and glutamate metabolism	5	2.13 E^{-2}	/	/	/	/	/	/	/	/	/	/
Pyruvate metabolism	/	/	4	1.62 E^{-2}	/	/	/	/	/	/	4	1.54 E^{-2}
Synthesis and degradation of ketone bodies	/	/	2	2.57 E^{-2}	/	/	/	/	/	/	/	/
Citrate cycle	/	/	/	/	/	/	/	/	4	8.16 E^{-3}	/	/
Biotin metabolism	/	/	/	/	/	/	/	/	/	/	2	1.55 E^{-2}
TOTAL	9 pathways		9 pathways		9 pathways		9 pathways		9 pathways		9 pathways	

Table S6: Metabolite set coverage highlighted by metabolites extracted in blood (CV<30%) according to the six sample preparations. The number of hits and the p-values are shown for each preparation by metabolite sets.

SALIVA

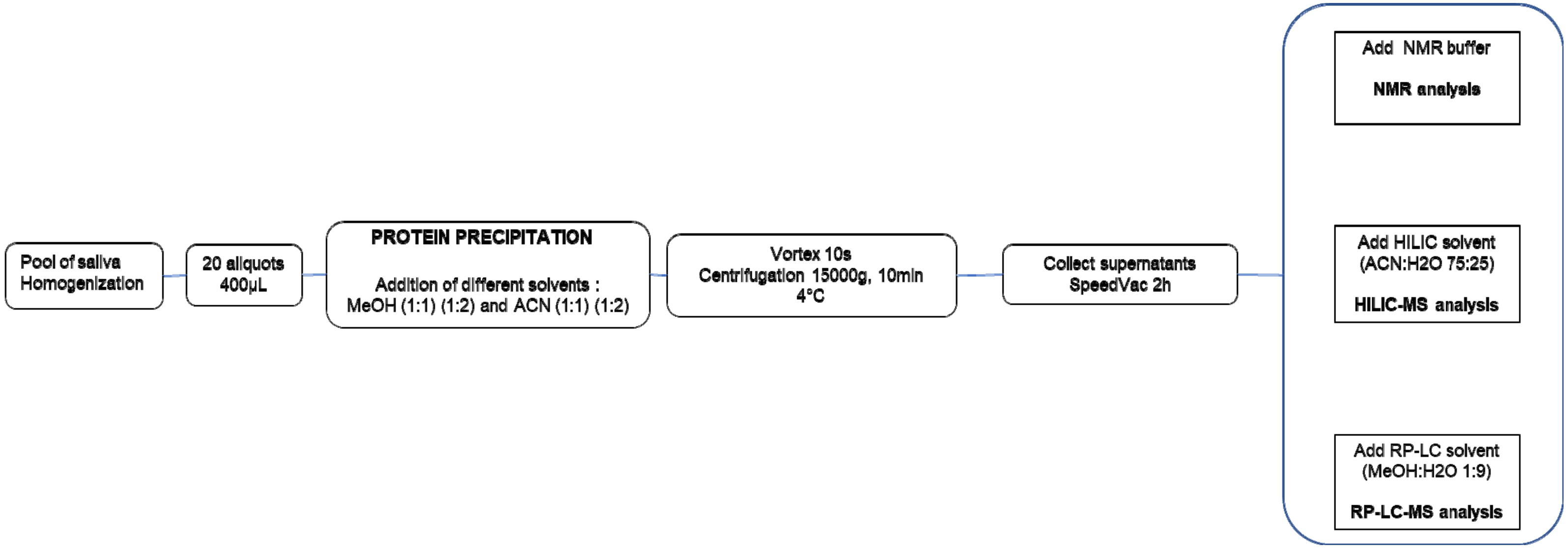

Figure S3: Schematic presentation of the sample preparation procedure applied on the pool of saliva for the selection of the optimum protocol for the maximum metabolome coverage by UPHLC-MS and ^{1}H-NMR

	Numbers of buckets					CV – standard deviation
	RP-LC POS	RP-LC NEG	HILIC POS	HILIC NEG	NMR	
Saliva :ACN (1 :2) (n=5)	987	520	1575	679	43	16% - 6%
Saliva :ACN (1 :1) (n=5)	988	684	1506	684	44	17% - 6%
Saliva :MeOH (1 :2) (n=5)	941	531	1233	725	47	19% - 6%
Saliva :MeOH (1 :1) (n=5)	987	407	1240	736	43	19% - 5%

Table S7: Number of extracted buckets in saliva for the different extraction methods in ^{1}H-NMR and UHPLC-MS

		RP-LC	HILIC	NMR	Total
Saliva :ACN (1 :2) (n=5)	Number of metabolites	53	108	18	147
	CV<5%	11	4	0	15
	CV<10%	38	43	9	77
	CV<20%	46	80	15	120
	**CV<30%**	**50**	**96**	**16**	**136**
	CV % mean (min-max)	11% (2%- 71%)	17% (3%-75%)	16% (8%-90%)	13% (2%-62%)
Saliva :ACN (1 :1) (n=5)	Number of metabolites	57	110	18	148
	CV<5%	18	3	7	28
	CV<10%	41	22	13	67
	CV<20%	50	63	15	110
	**CV<30%**	**53**	**98**	**16**	**137**
	CV % mean (min-max)	11% (1%- 78%)	20% (1%-80%)	11% (3%-54%)	14% (1%-80%)
Saliva :MeOH (1 :2) (n=5)	Number of metabolites	53	109	18	146
	CV<5%	13	5	2	20
	CV<10%	36	16	14	61
	CV<20%	46	49	16	96
	**CV<30%**	**50**	**90**	**18**	**133**
	CV % mean (min-max)	11% (2%- 65%)	21% (2%-80%)	8% (4%-28%)	15% (2%-80%)
Saliva :MeOH (1 :1) (n=5)	Number of metabolites	50	108	18	145
	CV<5%	22	19	0	39
	CV<10%	38	36	0	69
	CV<20%	47	68	14	111
	**CV<30%**	**49**	**89**	**16**	**133**
	CV % mean (min-max)	8% (1%- 50%)	19% (0%-93%)	22% (16%-52%)	15% (0%-93%)

Table S8: Number of extracted metabolites and coefficient of validation in saliva for the different extraction methods in ^{1}H-NMR and UHPLC-MS

Pathway name	ACN 1:2		ACN 1:1		MeOH 1:2		MeOH 1:1	
	Hits	*p*-value	Hits	*p*-value	Hits	*p*-value	Hits	*p*-value
Purine metabolism	14	1.98 E^{-5}	12	3.57 E^{-4}	14	1.98 E^{-5}	13	9.41 E^{-5}
Phenylalanine metabolism	5	1.62 E^{-4}	5	1.53 E^{-4}	5	1.62 E^{-4}	4	2.19 E^{-3}
Pyrimidine metabolism	9	4.40 E^{-4}	11	9.78 E^{-6}	10	7.72 E^{-5}	10	7.20 E^{-5}
Histidine metabolism	5	2.09 E^{-3}	/	/	6	2.33 E^{-4}	5	2.09 E^{-3}
Pantothenate and CoA biosynthesis	5	2.81 E^{-3}	5	2.66 E^{-3}	5	2.81 E^{-3}	5	2.81 E^{-3}
Glutathione metabolism	5	4.79 E^{-3}	/	/	7	7.64 E^{-5}	6	6.79 E^{-4}
Alanine, aspartate and glutamate metabolism	6	5.99 E^{-3}	7	1.05 E^{-3}	6	5.99 E^{-3}	7	1.13 E^{-3}
Glyoxylate and dicarboxylate metabolism	6	1.01 E^{-2}	7	2.01 E^{-3}	7	2.16 E^{-3}	8	3.87 E^{-4}
Glycerophospholipid metabolism	/	/	4	6.17 E^{-3}	/	/	/	/
Valine, leucine and isoleucine metabolism	/	/	3	9.99 E^{-3}	/	/	/	/
beta-Alanine metabolism	/	/	/	/	5	7.59 E^{-3}	/	/
TOTAL	8 pathways		8 pathways		9 pathways		8 pathways	

Table S9: Metabolite set coverage highlighted by metabolites extracted in saliva (CV<30%) according to the four sample preparations. The number of hits and the p-values are shown for each preparation by metabolite sets.

FECES

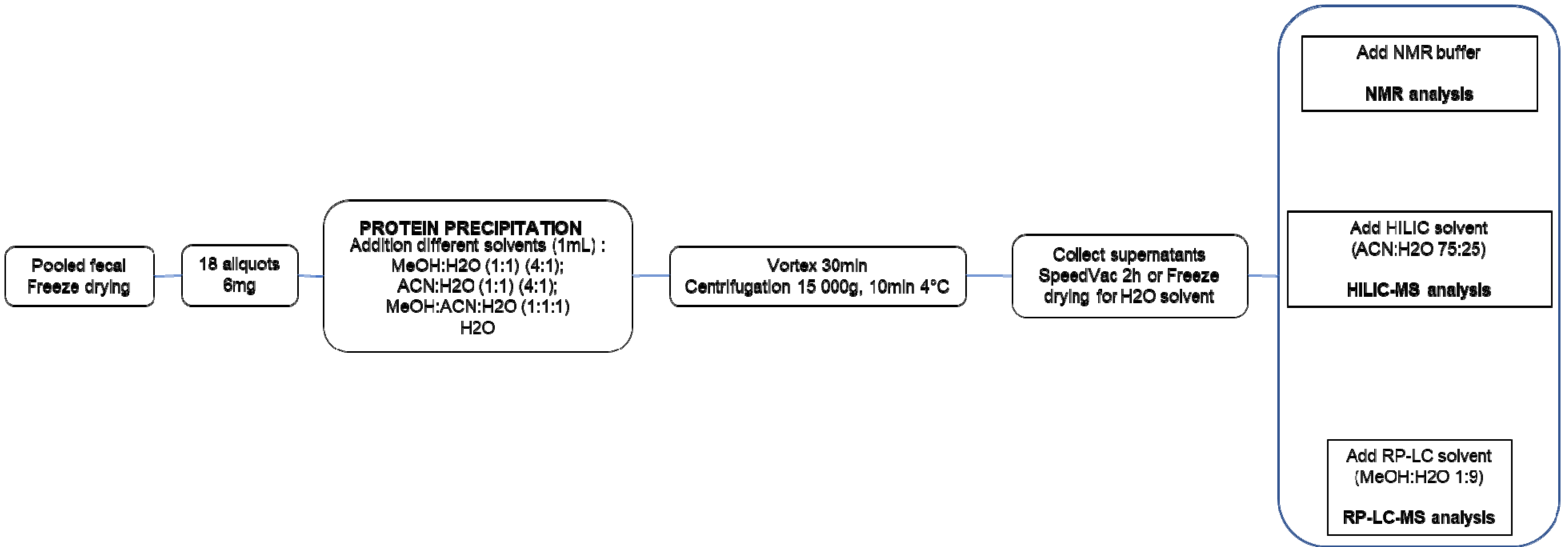

Figure S4: Schematic presentation of the sample preparation procedures applied in the pool of fecal samples for the selection of the optimum protocol for the maximum metabolome coverage by UHPLC-MS and ^{1}H-NMR.

	Numbers of buckets					CV – standard deviation
	**RP-LC POS**	**RP-LC NEG**	**HILIC POS**	**HILIC NEG**	**NMR**	
ACN:H_2O (1 :1) (n=5)	2013	1963	1948	1251	45	12% - 5%
ACN:H_2O (4 :1) (n=5)	1984	1931	1912	1261	45	10% - 5%
MeOH:H_2O (1:1) (n=5)	1908	1961	1851	1259	45	9% - 4%
MeOH :H_2O (4:1) (n=5)	1991	1953	1865	1187	45	10% - 5%
MeOH:H_2O:ACN (1 :1: 1) (n=5)	1827	1898	1863	1255	45	11% - 5%
H_2O (n=5)	1636	1737	1537	1043	45	12% - 6%

Table S10: Number of extracted buckets in feces for the different extraction methods in ^{1}H-NMR and UHPLC-MS

		RP-LC	HILIC	NMR	TOTAL
ACN:H_2O (1 :1) (n=5)	Number of metabolites	163	203	25	281
	CV<5%	1	28	0	29
	CV<10%	49	119	23	161
	CV<20%	128	174	25	250
	**CV<30%**	**145**	**192**	**25**	**266**
	CV % mean (min-max)	17% (1%-125%)	12% (1%-74%)	7% (6%-11%)	12% (1%-105%)
ACN:H_2O (4 :1) (n=5)	Number of metabolites	155	199	25	272
	CV<5%	17	30	4	47
	CV<10%	90	118	22	181
	CV<20%	126	172	25	241
	**CV<30%**	**140**	**189**	**25**	**259**
	CV % mean (min-max)	15% (2%-120%)	11% (2%-78%)	6% (4%-12%)	11% (2%-82%)
MeOH:H_2O (1:1) (n=5)	Number of metabolites	155	201	25	278
	CV<5%	26	41	0	63
	CV<10%	101	121	22	190
	CV<20%	129	171	25	242
	**CV<30%**	**143**	**190**	**25**	**264**
	CV % mean (min-max)	13% (2%-130%)	11% (2%-76%)	8% (5%-13%)	10% (2%-130%)
MeOH :H_2O (4:1) (n=5)	Number of metabolites	156	196	25	270
	CV<5%	62	24	9	79
	CV<10%	105	97	24	173
	CV<20%	136	166	25	246
	**CV<30%**	**143**	**184**	**25**	**259**
	CV % mean (min-max)	11% (1%-148%)	13% (2%-62%)	6% (3%-15%)	10% (1%-62%)
MeOH:H_2O:ACN (1 :1: 1) (n=5)	Number of metabolites	160	195	25	275
	CV<5%	19	35	8	55
	CV<10%	83	117	24	176
	CV<20%	127	160	25	236
	**CV<30%**	**141**	**184**	**25**	**258**
	CV % mean (min-max)	17% (2%-144%)	12% (2%-70%)	6% (3%-11%)	12%(2%-144%)
H_2O (n=5)	Number of metabolites	152	195	25	269
	CV<5%	27	9	4	36
	CV<10%	87	50	21	135
	CV<20%	131	127	25	218
	**CV<30%**	**140**	**173**	**25**	**254**
	CV % mean (min-max)	12% (2%-65%)	18% (2%-111%)	7% (4%-15%)	13%(2%-69%)

Table S11: Number of extracted metabolites and coefficient of validation in feces for the different extraction methods in ^{1}H-NMR and UHPLC-MS

Name	ACN:H2O 1:1		ACN:H2O 41		MeOH:H2O 1:1		MeOH:H2O 4:1		MeOH:H2O:ACN 1:1:1		H2O	
	**Hits**	***p*-value**	**Hits**	***p*-value**	**Hits**	***p*-value**	**Hits**	***p*-value**	**Hits**	***p*-value**	**Hits**	***p*-value**
Histidine metabolism	8	8.17 E^{-5}	9	9.02 E^{-6}	8	7.35 E^{-5}	9	6.64 E^{-6}	7	4.78 E^{-4}	8	5.91 E^{-5}
Tyrosine metabolism	12	8.52 E^{-4}	14	5.54 E^{-5}	13	1.82 E^{-4}	15	7.08 E^{-6}	12	5.27 E^{-4}	13	1.33 E^{-4}
Pyrimidine metabolism	13	8.95 E^{-5}	13	1.04 E^{-4}	13	7.66 E^{-5}	13	7.08 E^{-5}	14	9.60 E^{-6}	13	5.57 E^{-5}
Phenylalanine metabolism	6	1.89 E^{-4}	5	2.18 E^{-3}	5	1.91 E^{-3}	6	1.67 E^{-4}	5	1.61 E^{-3}	5	1.67 E^{-3}
Valine, leucine and isoleucine metabolism	6	3.03 E^{-5}	5	5.79 E^{-4}	6	2.78 E^{-5}	5	4.88 E^{-4}	6	2.23 E^{-5}	6	2.33 E^{-5}
Arginine and proline metabolism	11	9.80 E^{-4}	11	1.11 E^{-3}	11	8.65 E^{-4}	11	8.12 E^{-4}	11	6.26 E^{-4}	11	6.69 E^{-4}
Alanine, aspartate and glutamate metabolism	9	1.57 E^{-3}	9	1.74 E^{-3}	9	1.41 E^{-3}	9	1.34 E^{-3}	9	1.07 E^{-3}	9	1.14 E^{-3}
Tryptophan metabolism	12	6.69 E^{-4}	12	7.64 E^{-4}	12	5.84 E^{-4}	11	2.08 E^{-3}	11	1.62 E^{-3}	12	4.42 E^{-4}
Beta-Alanine metabolism	7	4.23 E^{-3}	7	4.59 E^{-3}	7	3.90 E^{-3}	7	3.74 E^{-3}	/	/	7	3.28 E^{-3}
Phenylalanine, tyrosine and tryptophane biosynthesis	/	/	3	4.51 E^{-3}	/	/	3	4.06 E^{-3}	/	/	/	/
Pantothenate and CoA biosynthesis	6	5.91 E^{-3}	6	6.35 E^{-3}	6	5.55 E^{-3}	6	5.30 E^{-3}	6	4.56 E^{-3}	6	4.74 E^{-3}
Glutathione metabolism	/	/	/	/	/	/	6	9.75 E^{-3}	6	8.43 E^{-3}	6	8.75 E^{-3}
Purine metabolism	14	6.11 E^{-3}	/	/	/	/	/	/	14	3.78 E^{-3}	/	/
Lysine degradation											6	8.75 E^{-3}
TOTAL	11 pathways		11 pathways		10 pathways		12 pathways		11 pathways		12 pathways	

Table S12: Metabolite set coverage highlighted by metabolites extracted in feces (CV<30%) according to the six sample preparations. The number of hits and the p-values are shown for each preparation by metabolite sets.

PLATFORM COMPLEMENTARITY

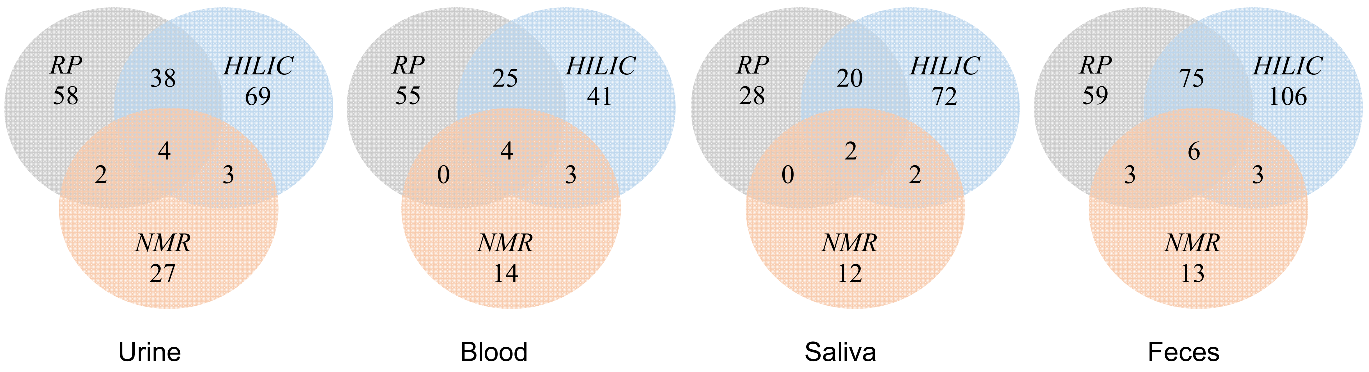

Figure S5: Venn diagram to mention the complementarity in term of metabolite coverage across the different analytical platforms according to the four matrices prepared with the chosen preparation

MATRICES COMPLEMENTARITY

Elements in FECES :		Elements in URINE :	Elements in SALIVA :	Elements in BLOOD :	Elements in FECES URINE :	Elements in BLOOD FECES :	Elements in FECES SALIVA :
**66 metabolites**		**25 metabolites**	**8 metabolites**	**6 metabolites**	**31 metabolites**	**12 metabolites**	**7 metabolites**
Methylglutaric acid	Imidazoleacetic acid	(2E,4E)-2,4-Hexadienoic acid	2-Ketobutyric acid	1-Methylhistidine	1-Naphthylamine	2-Hydroxy-3-methylbutyric acid	3-Hydroxyphenyllactate
(+/-)-2-Hydroxy-4-(methylthio)butanoic acid	Indole	17a-Estradiol	3-(3-Hydroxyphenyl)propanoic acid	2-Hydroxybutyric acid	2-Aminoisobutyric acid	3,4-Dihydroxybenzeneacetic acid	3-Sulfinoalanine
(R)-Salsolinol	Isovaleric acid	2-Methylglutaric acid	5-Methylcytosine	Dodecanoylcarnitine	3-(2-Hydroxyphenyl)propanoic acid	Glycocholic acid	Cadaverine
1-Aminocyclopropanecarboxylic acid	Ketoleucine	5-Hydroxy-L-tryptophan	Butanal	Propanal	3-Hydroxybenzoic acid	Homogentisic acid	Dethiobiotin
3-Amino-5-hydroxybenzoate	L-Acetylcarnitine	5'-Methylthioadenosine	Deoxyadenosine	Theobromine	3-Methoxytyramine	L-Glutamine	D-Xylose
3-Aminoisobutanoic acid	L-Asparagine	Acetoin	D-Ribose 5-phosphate	Thyroxine	3-Methyldioxyindole	L-Kynurenine	Histamine
4,5-Dihydroorotic acid	L-Dopa	Acetylcysteine	Propionic acid		4-Hydroxybenzoic acid	L-Proline	Malonate
4-Guanidinobutanoic acid	Lithocholic acid	Benzoic acid	Putrescine		CMPciliatine	MG(18:1(9Z)/0:0/0:0)	
4-Hydroxycinnamic acid	L-Leucine	Cinnamaldehyde			Dimethylamine	Protocatechuic acid	
4-Methylcatechol	L-Lysine	Citraconic acid			Ethylmalonic acid	Ribitol	
5-Aminopentanoic acid	Methylmalonic acid	Citric acid			Guanidoacetic acid	Salicyluric acid	
5-Hydroxyindoleacetic acid	Mevalonolactone	Cyclic GMP			Homocysteine thiolactone	Vanylglycol	
6-Mercaptopurine ribonucleoside triphosphate	N-Acetyl-L-methionine	Dimethylglycine			Homovanillic acid		
Adenosine monophosphate	N-Acetylputrescine	Galactaric acid			L-Arginine		
Adipic acid	N-Acetylserine	Glycolate			L-Glutamic acid		
Anserine	N-alpha-Acetyl-L-lysine	Hydroxyphenyllactic acid			L-Histidine		
Ascorbate	Nicotine	Indoxyl sulfate			L-Histidinol		
Benzyl alcohol	Normetanephrine	Itaconic acid			L-Serine		
Betaine	Oleic acid	L-Arabinose			Maleamate		
Butyric acid	Ornithine	Maleic acid			Melatonin		
Catechol	Orotic acid	Mannitol			N-Acetylneuraminic acid		
Citrulline	Palmitic acid	myo-Inositol			N-Methyltryptamine		
Cysteic acid	Phenylethylamine	Nicotinamide ribotide			O-Succinyl-L-homoserine		
Deoxycholic acid	Phosphorylcholine	Ophthalmic acid			Phenylacetaldehyde		
Deoxycytidine	p-Hydroxyphenylacetic acid	Tryptophanol			Pterin		
Diaminopimelic acid	p-Octopamine				Pyrrole-2-carboxylic acid		
Diethyl oxalpropionate	Purine				Shikimic acid		
D-Ribose	Pyridoxamine				Trigonelline		
Fumaric acid	Sarcosine				Ureidopropionic acid		
Gentisic acid	Tartaric acid				Valeric acid		
Heptanoic acid	Thiamine				Xanthurenic acid		
Hydroquinone	trans-Aconitic acid						
Hydroxykynurenine	Tryptamine						

**Elements in BLOOD URINE : 6 metabolites**	**Elements in BLOOD SALIVA : 2 metabolites**	**Elements in SALIVA URINE : 2 metabolites**	**Elements in BLOOD FECES URINE : 26 metabolites**	**Elements in FECES SALIVA URINE : 19 metabolites**	**Elements in BLOOD FECES SALIVA : 10 metabolites**	**Elements in BLOOD SALIVA URINE : 5 metabolites**	**Elements in BLOOD FECES SALIVA URINE : 37 metabolites**
Creatine	2-Aminophenol	AICAR	4-Acetamidobutanoic acid	1H-Indole-3-acetamide	3-Hydroxycapric acid	Caffeine	1-Methyladenosine
L-Malic acid	Phenylacetic acid	cis-Aconitic acid	4-Pyridoxic acid	3-Methyladenine	3-Methoxytyrosine	Choline	2-Hydroxypyridine
Quinic acid			4-Trimethylammoniobutanoic acid	Acetic acid	Caffeate	Isocitric acid	3-Hydroxyanthranilic acid
S-Adenosylhomocysteine			6-Hydroxynicotinic acid	Adenosine	Diethanolamine	N6-(delta2-Isopentenyl)-adenine	3-Hydroxybutyric acid
Sphinganine			Acetoacetic acid	Biotin	Leukotriene B4	N-Alpha-acetyllysine	3-Hydroxymethylglutaric acid
Trimethylamine N-oxide			All-trans-retinoic acid	Cytidine	L-Valine		Acetylglycine
			Azelaic acid	Deoxyuridine	Pectin		Adenine
			Cholic acid	Glycerophosphocholine	Pipecolic acid		Cortisol
			Cortisone	Guanosine	Theophylline		Creatinine
			Diacetyl	Inosine	Trimethylamine		Cytosine
			Ethanolamine	L-Aspartic acid			D-Glucose
			Indole-3-methyl acetate	N-Acetylglutamic acid			Dihydrouracil
			Indoleacetaldehyde	Nicotinic acid			Formic acid
			Kynurenic acid	Pimelic acid			Glycine
			L-Carnitine	Riboflavin			Guanine
			L-Lactic acid	Serotonin			Hippuric acid
			L-Threonine	Suberic acid			Hypoxanthine
			L-Tryptophan	Thymidine			Indoleacetic acid
			Lumichrome	Thymine			L-Alanine
			Methylguanidine				L-Isoleucine
			N-Acetyl-L-aspartic acid				L-Methionine
			N-acetyltryptophan				L-Phenylalanine
			Niacinamide				L-Tyrosine
			Quinaldic acid				Methyl jasmonate
			Taurine				N-Acetyl-L-alanine
			Uric acid				N-Acetylleucine
							N-Acetyl-L-phenylalanine
							N-Acetylserotonin
							Pantothenic acid
							Pyroglutamic acid
							Succinic acid
							trans-Ferulic acid
							Uracil
							Uridine
							Urocanic acid
							Xanthine
							Xanthosine

Table S13: Metabolites list link to the Upsetplot
